# Interactions between KSHV ORF57 and the novel human TREX proteins, CHTOP and CIP29

**DOI:** 10.1099/jgv.0.000503

**Published:** 2016-08

**Authors:** Sophie Schumann, Belinda Baquero-Perez, Adrian Whitehouse

**Affiliations:** ^1^​ School of Molecular and Cellular Biology, University of Leeds, Leeds, LS2 9JT, UK; ^2^​ Astbury Centre for Structural Molecular Biology, University of Leeds, Leeds, LS2 9JT, UK

**Keywords:** KSHV, hTREX, mRNA processing

## Abstract

The coupling of mRNA processing steps is essential for precise and efficient gene expression. The human transcription/export (hTREX) complex is a highly conserved multi-protein complex responsible for eukaryotic mRNA stability and nuclear export. We have previously shown that the Kaposi’s sarcoma-associated open reading frame 57 (ORF57) protein orchestrates the recruitment of the hTREX complex onto viral intronless mRNA, forming a stable and export-competent viral ribonucleoprotein particle (vRNP). Recently, additional cellular proteins, namely CHTOP, CIP29 and POLDIP3 have been proposed as novel hTREX components. Herein, we extend our previous research and provide evidence that ORF57 interacts with CHTOP and CIP29, in contrast to POLDIP3. Moreover, depletion studies show both CHTOP and CIP29 effect ORF57-mediated viral mRNA processing. As such, these results suggest both CHTOP and CIP29 are hTREX components and are recruited to an ORF57-mediated vRNP.

Eukaryotic mRNA biogenesis requires multiple post-transcriptional processing steps to be completed prior to nuclear export and translation. It is now evident that these mRNA processing steps, such as capping, splicing and polyadenylation, are carefully coordinated and mechanistically coupled (Brodsky & Silver, 2000; Lei & Silver, 2002; Zenklusen *et al.*, 2002). Failure to proceed through these steps leads to activation of the cellular mRNA surveillance machinery and subsequent mRNA decay (Fasken & Corbett, 2009; Hilleren *et al.*, 2001; Libri *et al.*, 2002). Nuclear replicating viruses have evolved multiple strategies to specifically recruit cellular RNA-binding proteins to enhance viral mRNA processing and nuclear export of viral mRNAs, which, in turn, increases virus gene expression and infectious virion production. We and others,have shown that herpesviruses utilize multiple components of the human transcription/export (hTREX) complex to stabilize and promote nuclear export of viral mRNAs (Schumann *et al.*, 2013). Specifically, the hTREX complex serves as a binding platform for Nxf1 on herpesvirus mRNAs, forming a stable and export-competent viral ribonucleoprotein particle (vRNP). Recruitment of the hTREX complex onto cellular mRNAs occurs in a splicing-dependent manner. In contrast, herpesviruses contain numerous intronless genes, and therefore rely on the presence of a viral adapter protein to recruit the hTREX complex onto these viral intronless mRNAs. The multifunctional and conserved open reading frame 57 (ORF57) gene product of Kaposi’s sarcoma-associated herpesvirus (KSHV) is one such adapter protein, specifically interacting with the hTREX complex in order to stabilize and subsequently export viral intronless mRNA, thereby enhancing viral protein production (Boyne *et al.*, 2008; Malik *et al.*, 2004; Nekorchuk *et al.*, 2007; Stubbs *et al.*, 2012).

Cellular hTREX is a large multi-protein complex; components include the RNA-helicase UAP56, which interacts directly with the mRNA export adapter Aly, which in turn binds the export receptor Nxf1 (Luo *et al.*, 2001; Zhou *et al.*, 2000). Alternatively, UIF also interacts with UAP56 and Nxf1, providing export adapter redundancy in the absence of Aly (Hautbergue *et al.*, 2009). The multiprotein sub-complex THO also associates with UAP56 and Aly to complete the central core components of hTREX. While THO is essential for the assembly of hTREX in yeast (Strässer *et al.*, 2002), surprisingly little is known about the role of mammalian THO. Additional cellular proteins CHTOP, CIP29 and POLDIP3 have been proposed as novel hTREX components (Chang *et al.*, 2013; Dufu *et al.*, 2010; Folco *et al.*, 2012). CHTOP and CIP29 were found to associate with UAP56 and other hTREX components in an ATP-dependent manner. While CHTOP binding to UAP56 could be outcompeted by Aly, CIP29 was shown to bind UAP56, forming a trimeric complex in conjunction with Aly (Dufu *et al.*, 2010). An ATP-dependent interaction with hTREX components has also been shown for POLDIP3; however, no specific interaction partner has been identified to date (Folco *et al.*, 2012).

We have previously shown that ORF57 recruits hTREX components Aly, UAP56 and the THO sub-complex onto viral intronless mRNA to facilitate vRNP formation (Boyne *et al.*, 2008; Jackson *et al.*, 2014). Furthermore, ORF57 is able to interact with UIF, providing adapter redundancy in the viral setting (Jackson *et al.*, 2011). Here, we extend our previous research and provide evidence that ORF57 also interacts with these putative hTREX components. This provides novel evidence that these cellular proteins may indeed be part of hTREX and also confirms that ORF57 recruits the complete hTREX complex onto viral mRNA in order to facilitate mRNA stability and export.

To examine the interaction of KSHV ORF57 with the putative hTREX components, HEK-293T cells were transfected with ORF57-eGFP or control eGFP for 24 h, Lipofectamine 2000 (Life Technologies). Cell lysates were then incubated with GFP-trap^®^ agarose beads (ChromoTek^®^), as previously reported (Jackson *et al.*, 2011) to precipitate protein complexes associated with ORF57 or eGFP. RNase A (ThermoFisher Scientific) (0.01 U ml^−1^) was added to all lysates to disrupt RNA bridging, unless otherwise indicated. Fig. 1 shows Western blots of the precipitated proteins analysed using GFP- (Clontech), CHTOP- (Bethyl Laboratories, Inc.), CIP29- (serum previously described in (Dufu *et al.*, 2010) and POLDIP3- (Bethyl Laboratories, Inc.) specific antibodies. Both CHTOP and CIP29 specifically interacted with ORF57-eGFP, but not with control eGFP protein (Fig. 1a, 1b). Surprisingly, however, POLDIP3, which was seen expressed as both α- (46 kDa) and β-splice variant (43 kDa), did not co-precipitate with ORF57 in the presence of RNase A. We therefore repeated the immunoprecipitations, both in the absence and presence of RNase A, and found that in contrast to CHTOP and CIP29, POLDIP3 appears to interact with ORF57 only in the absence of RNase A (Fig. 1c). This indicates an indirect interaction of the two proteins, which is most likely bridged by RNA or other RNA-binding proteins.

**Fig. 1.**
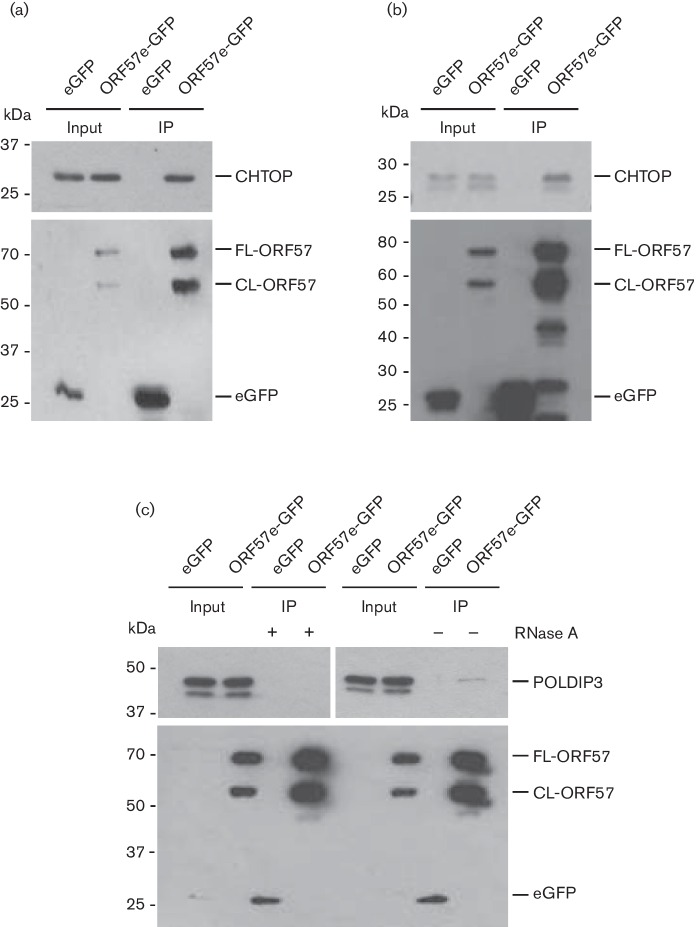
KSHV ORF57 interacts with hTREX components CHTOP, CIP29 and POLDIP3. Cells were transfected with eGFP or ORF57-eGFP and co-immunoprecipitations performed using GFP-affinity beads. RNase A was added to all whole-cell lysates, unless otherwise stated. Whole-cell lysates (input) and precipitations were analysed by Western blot using GFP-specific and (a) CHTOP-specific, (b) CIP29-specific and (c) POLDIP3-specific antibodies. FL- and CL-ORF57 denotes either full-length (FL) ORF57 or the caspase-7 cleavage product (CL).

To confirm these results, immunofluorescence was utilized to examine the subcellular localization of ORF57 and the novel hTREX components. ORF57 is a nucleocytoplasmic protein that is usually observed in the nucleolus and nuclear splicing speckles (Boyne *et al.*, 2008; Jackson *et al.*, 2011. Cells were transfected for 24 h with eGFP-, or ORF57-eGFP-expression plasmids and immunofluorescence was performed with a CIP29-specific antibody (Fig. 2a). Endogenous CIP29 was found to localize to nuclear splicing speckles and to exclude the nucleolus in the presence of eGFP (co-staining with markers for splicing speckles and the nucleolus shown in Fig. S1a, S1b, available in the online Supplementary Material). In contrast, in the presence of ORF57-eGFP, CIP29 was recruited into the nucleolus (arrow), where it co-localized with ORF57-eGFP, losing most of its speckled appearance. As endogenous antibodies for CHTOP and POLDIP3 were found unsuitable for immunofluorescence, we overexpressed fusion proteins of CHTOP and POLDIP3 in the absence or presence of ORF57. CHTOP-eGFP was co-expressed with control mCherry or mCherry-ORF57, while POLDIP3-HA was co-expressed with control eGFP or ORF57-eGFP. The fusion proteins tagged to eGFP and mCherry were visualized through direct fluorescence, whereas immunofluorescence using specific HA-antibody was utilized to visualize POLDIP3. Fig. 2(b) and 2(c) shows co-localization of CHTOP and POLDIP3 with ORF57. While CHTOP-eGFP was observed diffusely throughout the nucleus and predominantly located in the nucleolus when co-expressed with mCherry, in the presence of ORF57, localization appeared more punctate and in nuclear splicing speckles (dashed arrows) (Fig. S1a). In addition, CHTOP also co-localized with ORF57 in the nucleolus (arrows) (Fig. S1b). In contrast to CHTOP and CIP29, POLDIP3-HA failed to re-localize from nuclear splicing speckles into the nucleolus upon ORF57-eGFP co-expression. Here, ORF57 was redistributed from mainly a nucleolar localization to co-localize with POLDIP3 in foci, which partially co-stains with the nuclear speckle marker, Sc35 (Fig. S1a).

**Fig. 2.**
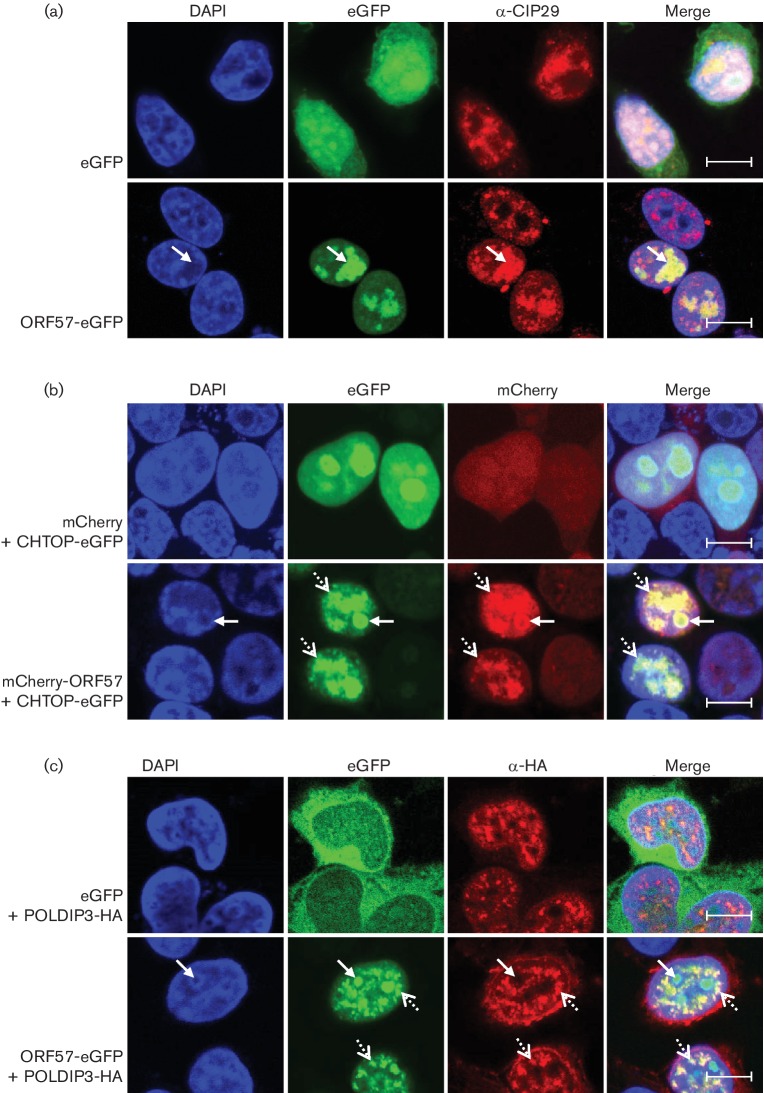
ORF57 recruits CHTOP and CIP29, but not POLDIP3, into the nucleolus. (a) Confocal microscopy of cells expressing eGFP or ORF57-eGFP. Cells were stained using a CIP29-specific antibody and the nucleus visualized using DAPI. Arrows indicate the nucleolus. Scale bar, 10 µm. (b) Confocal microscopy of cells expressing the indicated expression plasmids of ORF57 and CHTOP or POLDIP3, respectively. (c) Where appropriate, cells were stained using a HA-specific antibody and fixed using methanol, while all other cells were fixed using formaldehyde. The nucleus was visualized using DAPI. Arrows indicate the nucleolus, dashed arrows indicate nuclear speckles. Scale bar, 10 µm.

ORF57 is a multifunctional protein influencing many aspects of viral mRNA processing, interacting with hTREX components to stabilize and enhance viral intronless mRNA export. We therefore performed mRNA export assays using a viral reporter mRNA as previously described (Jackson *et al.*, 2011), in cells that were specifically depleted for each hTREX component. Cells were transfected in 12-well plates with 100 nM siRNA (final siRNA concentration of the cells) as previously described (Baquero-Pérez & Whitehouse, 2015). For POLDIP3, a pool of three target-specific siRNAs (Santa Cruz) and for SRAG and CIP29 a siGENOME SMARTpool of 4 siRNAs (Dharmacon) was used. To assess optimal protein depletion, cell lysates were collected every 24 h post-siRNA transfection over a 72 h period. Immunoblotting was performed using CHTOP-, CIP29- or POLDIP3-specific antibodies, as well as a GAPDH-specific antibody as loading control as previously described (Hughes *et al.*, 2015. Fig. 3(a–c) shows depletion of all three putative hTREX components over 72 h. While complete knock-down was not observed for CHTOP, CIP29 or POLDIP3, a marked decrease (>50 %) in all protein levels was observed at 72 h. For mRNA export assays, cells were transfected with CIP29-, CHTOP- or POLDIP3-specific siRNA or scramble control for 72 h, to allow sufficient protein depletion. After 72 h, cells were transfected again with eGFP- or ORF57-eGFP-expression plasmids, as well as a viral intronless reporter mRNA (ORF47). After 24 h, cells were fractionated into whole-cell and cytoplasmic fractions through lysis for 10 min in PBS + 1 % Triton, and subsequent centrifugation at 2000****g**** for 5 min. ORF47-mRNA levels were quantified by qRT-PCR and normalized to GAPDH levels. Fig. 3(d–f) displays the changes in cytoplasmic and whole-cell levels of the viral reporter mRNA in the absence and presence of the indicated hTREX components and ORF57. As previously described, the presence of ORF57 causes stabilization and accumulation of viral intronless mRNA (Nekorchuk *et al.*, 2007) and hence leads to increased whole-cell amounts of the viral reporter transcript, which enhances ORF57-mediated nuclear export and increased cytoplasmic levels of the reporter gene. CHTOP depletion decreased both whole-cell and cytoplasmic levels of the viral reporter mRNA in the presence of ORF57 at a similar ratio (Fig. 3d), suggesting a role of CHTOP in vRNP formation and primarily viral mRNA stability which affects downstream export. Surprisingly, CIP29 depletion led to a significant increase in whole-cell ORF47 mRNA levels by almost twofold (Fig. 3e). Similarly, cytoplasmic levels were also increased, indicating that loss of CIP29 does not negatively affect ORF57 function. Interestingly, the ratio of whole-cell to cytoplasmic levels of ORF47 transcript remained identical between depleted and CIP29-expressing cells (1.5-fold). This indicates that while CIP29 knock-down enhances the levels of ORF47 transcripts in the presence of ORF57, mRNA export is not specifically enhanced. While two opposing effects were seen following depletion of CHTOP or CIP29, no significant change was observed in ORF57-dependent viral mRNA stability and export in POLDIP3-depleted cells. Both whole-cell and cytoplasmic levels of viral intronless mRNA remain unchanged in the absence or presence of this putative hTREX component. These data suggest that POLDIP3 may not be involved in ORF57-mediated viral mRNA processing.

**Fig. 3.**
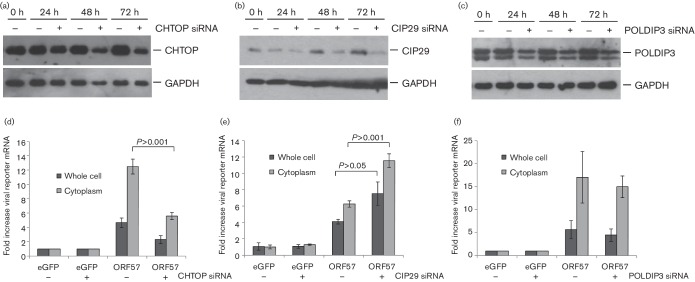
Depletion of CHTOP and CIP29, but not POLDIP3, affects ORF57-mediated viral mRNA processing . (a–c) siRNA-mediated knock-down of CHTOP, CIP29 or POLDIP3 over 72 h was analysed by Western blot, using the indicated antibodies. (d–f) Cells were transfected with ORF57-eGFP or eGFP and an ORF47 reporter construct 48 h after knock-down of CHTOP, CIP29 and POLDIP3 (as indicated). 24 h post-transfection, RNA was isolated from total and cytoplasmic fractions and levels were determined by qRT-PCR. Fold increase was determined using the ΔΔCT method with GAPDH as internal reference. Value are averages of two (d) or three (e, f) biological repeats, error bars present sd. *P* values were determined using Student’s **t**-test.

Together, we have examined the interaction of three novel, putative hTREX components, CHTOP, CIP29 and POLDIP3, and their role in stability and processing of viral RNAs. The endogenous protein CHTOP is loaded onto hTREX in an ATP-dependent manner by UAP56, and has been shown to subsequently provide a landing platform for the cellular export factor Nxf1, in order to facilitate mRNA nuclear export (Chang *et al.*, 2013). Here, we used co-immunoprecipitations and confocal microscopy to demonstrate that ORF57 interacts with CHTOP. Furthermore, we employed a viral mRNA export assay to confirm that CHTOP is an essential component of the vRNP. Following CHTOP protein depletion, ORF57-mediated stability and export of a viral intronless mRNA showed a more than twofold reduction. As previously demonstrated, depletion of CHTOP results in deficient cellular bulk mRNA export (Chang *et al.*, 2013). Similarly, we could observe the export block imposed on ORF57-dependent export of viral intronless mRNA, confirming that the role of CHTOP in hTREX assembly is also essential in the viral setting. Furthermore, whole-cell amounts of the viral reporter mRNA were also decreased. This is expected, as depletion of CHTOP is likely to result in an incomplete vRNP, which may affect the ability of ORF57 to stabilize and subsequently export intronless viral mRNAs.

CIP29 interacts with key components of the hTREX complex in an ATP-dependent manner, where it forms a trimeric sub-complex (Dufu *et al.*, 2010). Again, we showed that ORF57 interacts with CIP29 to form a vRNP, using co-immunoprecipitation and confocal microscopy. Surprisingly, however, viral mRNA stability and export were not reduced during CIP29 depletion. In contrast, whole-cell levels, as well as cytoplasmic levels of viral intronless mRNA were increased. It has previously been described that depletion of individual hTREX proteins can induce overexpression of other hTREX components in order to rescue the phenotype and provide a functional hTREX complex (Hautbergue *et al.*, 2009; Viphakone *et al.*, 2012). In the absence of CIP29, we observed overexpression for CHTOP, Thoc1 and Thoc5 (Fig. S2). Knock-down of potentially redundant CIP29 could therefore lead to overexpression of essential export factors, such as CHTOP, which would then promote higher transcript stability and nuclear export rates, as observed here. We therefore speculate that redundancy might exist for CIP29, as previously observed for Aly, both on a cellular and viral level (Hautbergue *et al.*, 2009; Jackson *et al.*, 2011).

Finally, we examined the interaction of POLDIP3 with ORF57. While POLDIP3 was also reported to interact with other hTREX components in an ATP-dependent manner, no direct interaction partner, nor the role of POLDIP3 within hTREX, has been elucidated (Folco *et al.*, 2012). Here, we performed co-immunoprecipitations with ORF57-GFP and found that POLDIP3 only co-precipitated in an RNA-dependent manner. This suggests the lack of a direct protein–protein interaction between the ORF57/hTREX complex and POLDIP3, and instead, bridging by RNA or other RNA-binding proteins. Furthermore, while overexpressed POLDIP3 was found in nuclear splicing speckles, as previously described (Folco *et al.*, 2012), we did not observe any relocalization upon co-expression with ORF57. ORF57 contains three nuclear localization signals (NLSs), two of which act together as a nucleolar localization signal (Boyne & Whitehouse, 2006 , 2009). While the role of the nucleolar localization is yet to be elucidated, it appears necessary for ORF57-mediated viral mRNA processing (Majerciak* et al*., 2006). Accordingly, ORF57 co-localizes with all hTREX components within the nucleolus (Jackson *et al.*, 2012). In contrast, our immunofluorescence data suggest that it is in nuclear splicing speckles, a compartment enriched in unspliced mRNAs and pre-mRNA splicing factors, where ORF57-GFP and POLDIP3 interact. Additionally, we found that knock-down of POLDIP3 had no significant effect on viral intronless mRNA stability or export. Interestingly, while it has been shown that POLDIP3 overexpression causes a block in cellular bulk mRNA export, no data have been published showing an effect for POLDIP3 knock-down (Folco *et al.*, 2012). While our results could be attributed to insufficient protein knock-down, taken in combination with the other data herein, we speculate that POLDIP3 is not part of the ORF57-mediated vRNP and does not play a crucial role in viral RNA processing. Previously, due to their spatial and functional closeness, components of hTREX have been mistakenly described as part of the exon-junction complex (EJC) (Le Hir *et al.*, 2001). While both complexes appear distinct in location and function, both are essential for correct cellular mRNA processing and appear to be dynamically remodelled. Interestingly, ORF57 has been shown to specifically interact with the EJC component, PYM, to enhance viral mRNA translation (Boyne *et al.*, 2010). It can therefore be imagined that POLDIP3 is a functional part of the EJC and further investigations should be undertaken.

Together, we have shown that ORF57 interacts with the CHTOP and CIP29, by co-immunoprecipitation and confocal microscopy. Moreover, viral mRNA export assays showed opposing effects following their depletion on ORF57-facilitated stability and export of viral intronless mRNA. In contrast, we speculate that the POLDIP3 is not part of the ORF57-mediated vRNP. As such, these results add further support to CHTOP and CIP29 being hTREX components.

## Supplementary Data

495 Supplementary File 1
