# Supplementary material for: Interactions between KSHV ORF57 and the novel human TREX proteins, CHTOP and CIP29
Source: J Gen Virol. 2016 Aug;97(8):1904–10. doi: 10.1099/jgv.0.000503 (PMC5156329; doi:10.1099/jgv.0.000503)
Supplement: Supplementary file 1 [file jgv-97-1904-s001.pdf]

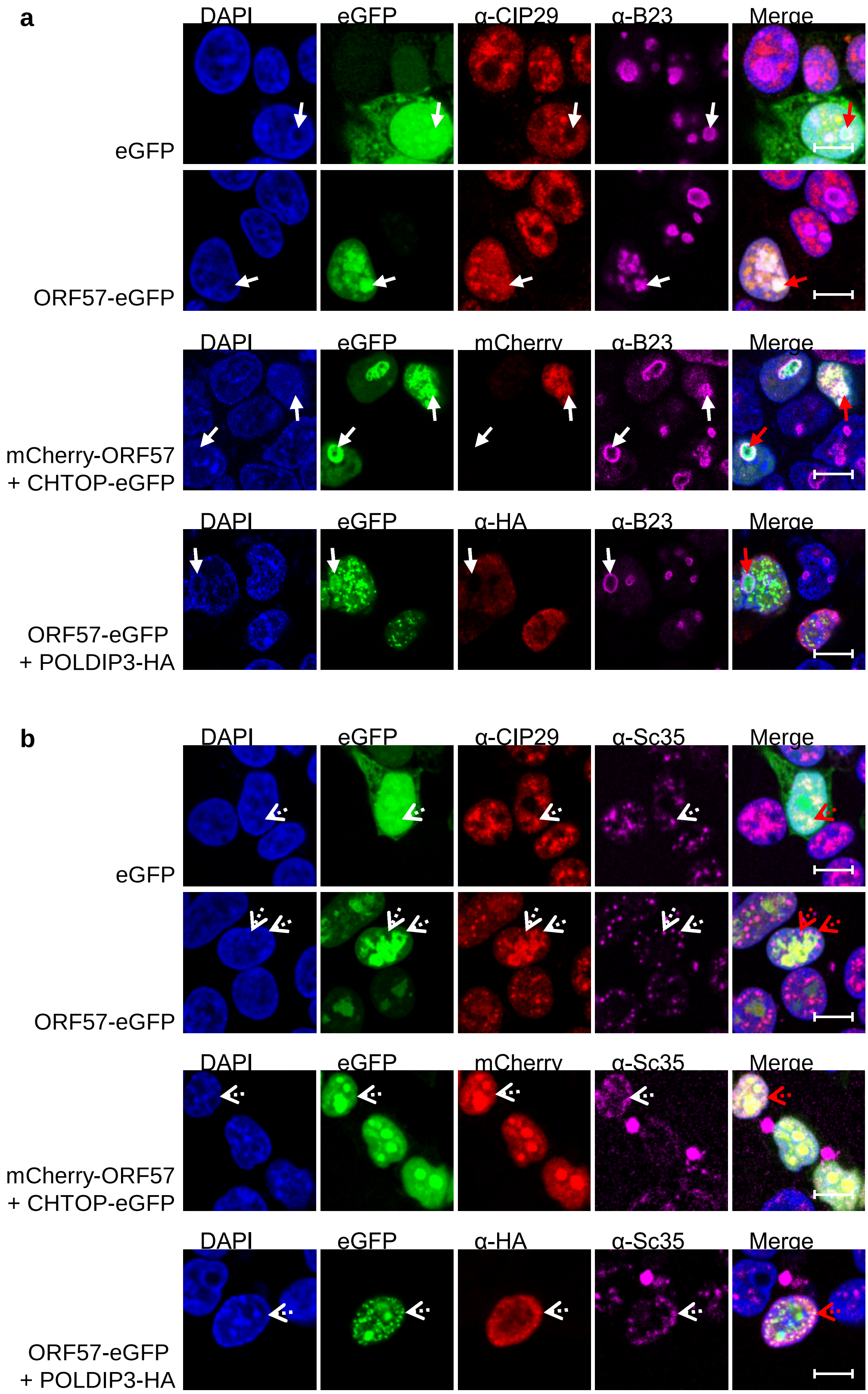

Supplementary Fig. 1: Co-staining of ORF57 and CHTOP, CIP29 or POLDIP3 with cellular markers Sc35 and B23.

(a) Confocal microscopy of cells expressing the indicated expression plasmids of eGFP, ORF57, CHTOP or POLDIP3, respectively. Cells were stained using a CIP29- or HA-specific antibody where indicated, as well as Sc35-specific antibody to stain nuclear splicing speckles. The nucleus was visualised using DAPI. Dashed arrows indicate nuclear splicing speckles. Scale bar = 10 µm. (b) Confocal microscopy of cells expressing the indicated expression plasmids of eGFP, ORF57, CHTOP or POLDIP3, respectively. Cells were stained using a CIP29- or HA-specific antibody where indicated, as well as a B23-specific antibody to stain nucleoli. The nucleus was visualised using DAPI. Arrows indicate the nucleus. Scale bar = 10 µm. (a, b) All cells were fixed with formaldehyde in order to visualise subcellular components using Sc35- and B23-specific antibodies, however, this was found to impair staining of the HA-antibody to visualise POLDIP3.

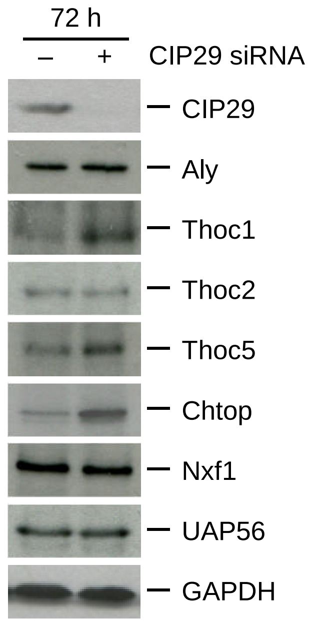

Supplementary Fig. 2: CIP29 depletion leads to differential expression of certain hTREX proteins. Immunoblotting was performed on control and CIP29 depletied cells using a wide range of hTREX-specific antibodies. Results show that CIP29 depletion leads to enhanced expression of Chtop, Thoc2 and Thoc5.
